# Short-term outcomes of laparoscopic intersphincteric resection with intraoperative radiotherapy using low-energy X-rays for primary locally advanced low rectal cancer: a single center experience

**DOI:** 10.1186/s12957-020-1799-x

**Published:** 2020-02-03

**Authors:** Wangsheng Xue, Shuang Wang, Zeyun Zhao, Yongbo Li, An Shang, Donglin Li, Jianzheng Yang, Tiejun Wang, Min Wang

**Affiliations:** 1 Department of the General Surgery, The Second Hospital of Jilin University, Changchun, Jilin Province China; 2 Department of Dermatology, The Second Hospital of Jilin University, Changchun, Jilin Province China; 3 Department of Radiology, The Second Hospital of Jilin University, Changchun, Jilin Province China

**Keywords:** Lap ISR, Low-energy X-rays, IORT, T3N0M0, T1–3N+M0, Low rectal cancer

## Abstract

**Background:**

Even with the augmentative application of anal-preservation surgery in low rectal cancer, the role and indications of laparoscopic intersphincteric resection (Lap ISR) are still under debate, especially for T3 or node-positive (T3N0M0, T1–3N+M0) cancer, mainly due to the oncological safety and functional outcomes. INTRABEAM (Carl Zeiss, Germany) intraoperative radiotherapy (IORT) using low-energy X-rays features in accurate irradiation, less exposure, and reduced complications. Taking advantages of Lap ISR and INTRABEAM IORT, this innovative approach aims to increase the probability of the anal preservation with acceptable postoperative outcomes.

**Materials and methods:**

From December 2015 to August 2019, we retrospectively analyzed the short-term outcomes of 12 patients evaluated preoperatively with T3 or node-positive (T3N0M0, T1–3N+M0) primary locally advanced low rectal cancer. They all had received Lap ISR and INTRABEAM IORT with a dose of 16–18 Gy applied by an applicator through the anus (natural orifice). Then, with no pre- or postoperative radiotherapy given, the patients were suggested to receive 6–8 cycles of the XELOX chemotherapy regimen (oxaliplatin, 130 mg/m^2^ and capecitabine, 1000 mg/m^2^).

**Results:**

All patients achieved R0 resection. The median radiation time was 27 min and 15 s, and the mean radiative dose was 17.3 Gy (range 16–18 Gy). The median follow-up time was 18.5 months (range 3–45 months). Two patients experienced local recurrence. Two male patients experienced anastomotic stenosis. Furthermore, one of them experienced perianal abscess and the other one experienced pulmonary metastasis after refusing to receive chemotherapy. One female patient with internal anal sphincter invasion experienced distant metastases to the liver and gluteus maximus muscle 35 months after IORT. No acute radiation injuries or symptoms were observed. Although they experienced a reduction in anal function, every patient was satisfied with the postoperative outcomes.

**Conclusions:**

For patients evaluated preoperatively with T3 or node-positive (T3N0M0, T1–3N+M0) primary locally advanced low rectal cancer, Lap ISR with INTRABEAM IORT may be a safe and feasible approach for anal preservation without compromising oncological outcomes.

## Introduction

Colorectal adenocarcinoma is the third most common cancer worldwide, and low rectal cancer refers to those the lower margin of a cancerous lesion located less than 5 cm from the anal verge. Distal resection margin (DRM) and circumferential resection margin (CRM) are both closely associated with local recurrence (LR) and disease-free survival (DFS) [1, 2]. Traditionally, due to the limited width of the distal pelvis and the 5-cm rule of the surgical distal-free resection margin, abdominoperineal resection (APR) is usually performed, which results in poor quality of life and the mental and psychological trauma to patients.

Recently, with the proposal of the 2-cm or even 1-cm rule [3] of the surgical distal-free margin and with the development of minimally invasive technology, laparoscopic intersphincteric resection (Lap ISR), defined as a laparoscopy-assisted surgical procedure specifically for internal anal sphincter (IAS) removal followed by hand-sewn colon-anal anastomosis without mucosectomy, and aiming to save the anus, has attracted attention. However, the criteria for indications and contraindications of Lap ISR have not reached a consensus. Commonly, patients preoperatively diagnosed with T1 or T2 or with node-negative cancer are selected, and the presence of T4 cancer, regardless of whether the cancer is node-negative, is considered a contraindication.

Hence, whether T3 or node-positive (T3N0M0, T1–3N+M0) cancer patients are suitable for Lap ISR is under debate. To achieve a good oncological outcome, T3 or node-positive (T3N0M0, T1–3N+M0) patients are commonly suggested to receive neoadjuvant chemotherapy with preoperative external beam radiotherapy (EBRT) to reach downstage to achieve better DRM and CRM. Although EBRT has been found to be effective to the pelvis, it may result in injury to adjacent normal structures, affecting the recovery of sphincter muscles [4]. Furthermore, long-course radiotherapy delays the time of surgery to some extent [5]. Short-course radiotherapy has little effect on improving the rate of anal preservation because it is difficult for tumors to achieve full atrophy due to the short interval [6].

Currently, to improve the LR and avoid the risks related to EBRT, the addition of intraoperative radiotherapy (IORT), defined as a directly single higher dose of irradiation to a tumor bed, a residual neoplasm, or an area of lymphatic drainage during surgery, has been widely used [7–9]. Compared with EBRT, IORT has advantages such as the potential for dose escalation, a reduced overall treatment time, and increased patient convenience. In particular, the main advantage of IORT is sterilizing close or positive resection margins.

Traditionally, IORT has included intraoperative electron radiation therapy (IOERT) and intraoperative high-dose rate brachytherapy (HDR-IORT). However, IOERT must be delivered in special shielded operating rooms [10]. The dose of HDR-IORT at the surface is higher than that of IOERT [11]. Compared with IOERT, the INTRABEAM photon radiosurgery system (PRS) (Carl Zeiss, Germany), which emits low-energy (50 kV) photons at a high-dose rate and modulates the electron beam to soft X-rays in a uniform dose [12], has been recommended for use in breast cancer by National Institute for Health and Care Excellence (NICE) [13] and has been performed in other tumors of the brain, rectum, and bone as a novel addition for improving LR [8, 12].

**Fig. 1:**
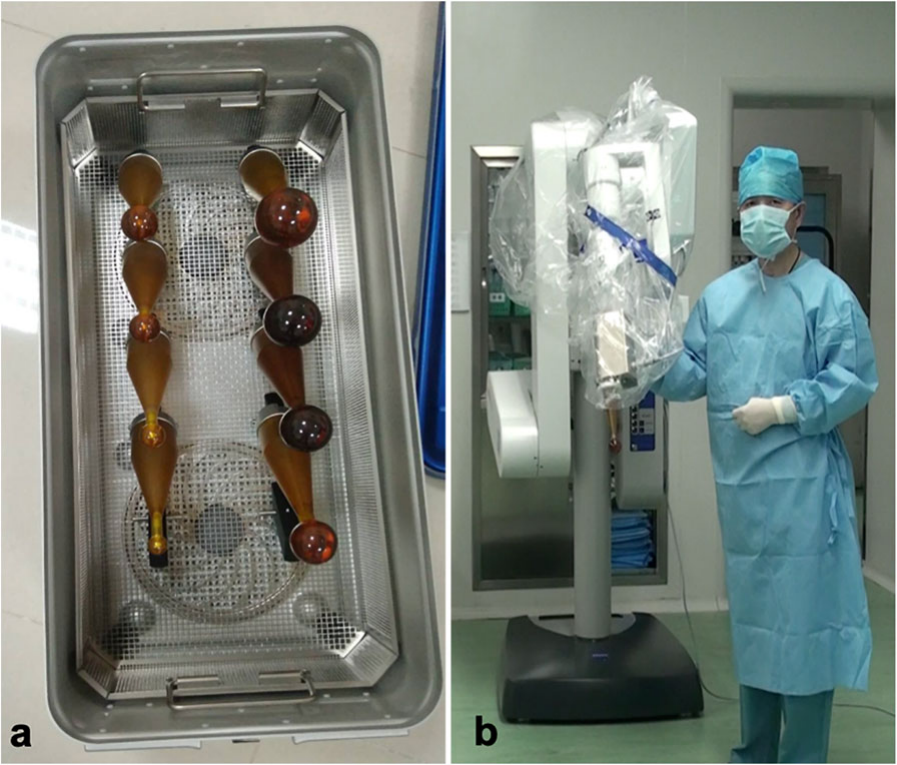
The INTRABEAM PRS device. **a** The different sizes of spherical applicator. **b** The appearance of INTRABEAM PRS device

Compared with IOERT and HDR-IORT, INTRABEAM PRS (Fig. 1b) can generate a homogenous dose distribution on the spherical applicator surface with rapid dose attenuation from the applicator (Fig. 1a) to the surface of the targeted site, contributing to better local control and reducing damage to the adjacent critical tissues. Furthermore, the applicator, with the flexibility at 6 degrees [14] of freedom, can be pushed into the targeted area transanally. Therefore, combining the advantages of Lap ISR and INTRABEAM IORT, this pilot study provides a new treatment modality for preserving the anus and improving the LR in locally advanced low rectal cancer. After reviewing the literature, we determined that the combination of the two therapies is completely novel, and we first presented the short-term outcomes of feasibility and safety herein.

## Materials and methods

The current study complied with the Declaration of Helsinki and was approved by the Ethics Committee of the Second Hospital of Jilin University with informed consent from each patient. This interdisciplinary approach—Lap ISR with INTRABEAM IORT—was carried out by the same team of surgeons, radiation oncologists, and technicians.

### Patient selection

From December 2015 to August 2019, 12 patients with a strong desire to preserve the anus were uninterruptedly registered in the study. Inclusion criteria were as follows: patients preoperatively diagnosed with T3 or node-positive (T3N0M0, T1–3N+M0) primary locally advanced low rectal cancer by MRI or ultrasonic endoscopy, the lower edge of the tumor was less than 5 cm from the anal edge or less than 3 cm from the dentate line, adequate preoperative sphincter function and continence, well or moderately differentiated rectal cancer according to biopsy specimens, absence of distant metastases, and strong desire to achieve anal preservation. Exclusion criteria were as follows: age > 85 years, low differentiated or undifferentiated adenocarcinoma, and had received preoperative radiotherapy.

### Surgical techniques

**Fig. 2:**
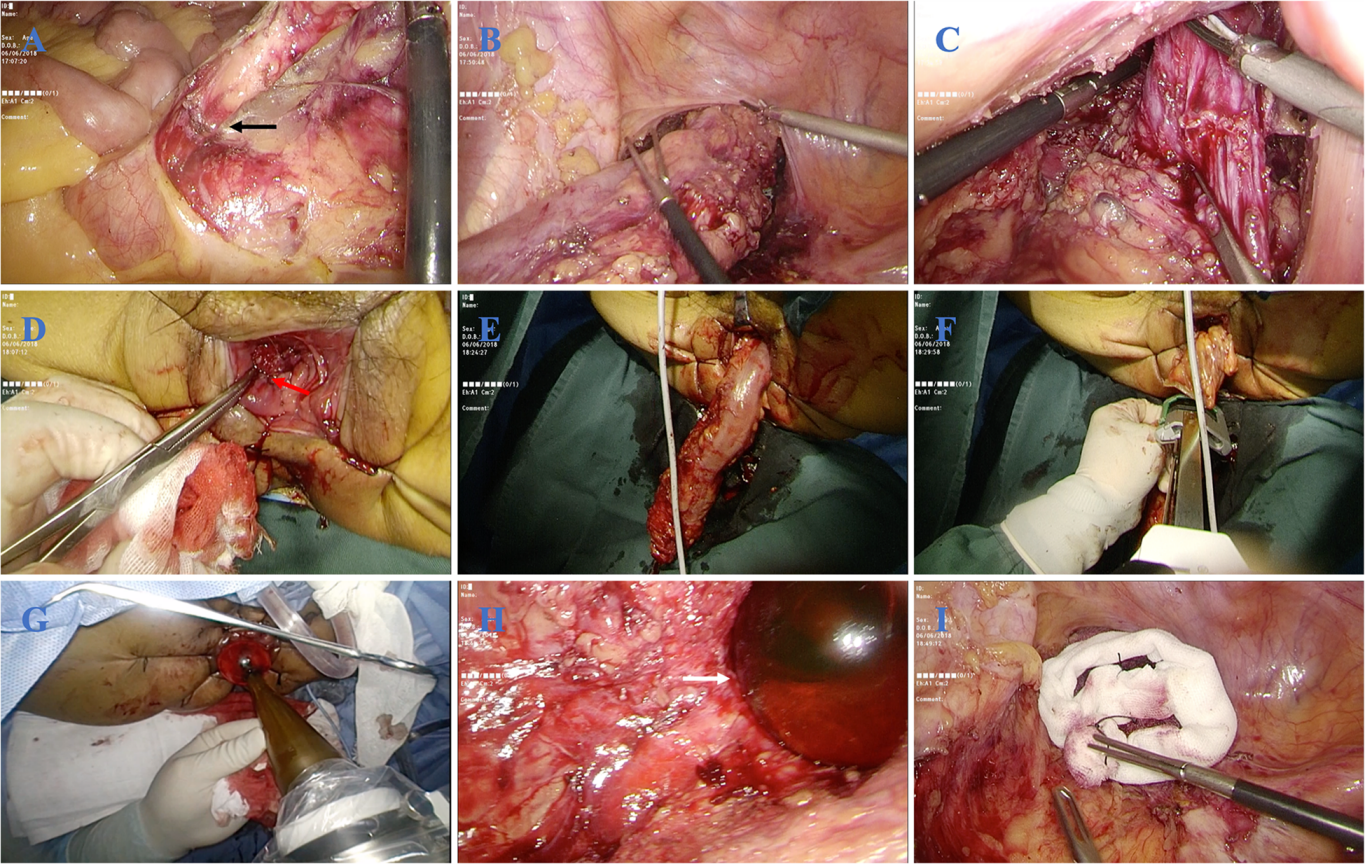
Main steps of surgery. **a** IMA was exposed and then ligated. Black arrow indicates the root of IMA. **b** The dissection of mesorectum in transabdominal approach. **c** The dissection to intersphincteric groove in transabdominal approach. **d** Purse-string suture was performed to expose the anus to achieve the optimal view in transanal approach. Red arrow indicates the lower edge of the tumor. **e** The specimen was dragged out by anus. **f** The tumor specimen was dissected by the linear stapler. **g** The applicator was pushed into the tumor bed transanally. **h** The applicator was put into the tumor bed by laparoscopic surveillance. White arrow points the spherical applicator in the pelvic cavity. **i** Wet gauzes were put to isolate and protect the adjacent structures from radiation

1. When the patient was under general anesthesia and was placed in a lithotomy position, laparoscopic exploration was performed after the pneumoperitoneum was established.
2. The patient position was transferred to a right-head-ventral side position so that the ileum could be removed to expose the left side of the colon.
3. During the laparoscopic procedure, the origin of the inferior mesenteric artery (IMA) was ligated, and lymphadenectomy was performed around the artery (Fig. 2a).
4. Following the principles of TME, the left side of the colon was dissected to the splenic flexure of the colon. The hypogastric nerves were identified to maintain protection and the rectum was mobilized to facilitate the transanal approach (Fig. 2b).
5. When dissection progressed to the endopelvic fascia and levator ani muscle (Fig. 2c), the transanal approach was operated.
6. During the transanal procedure, the anal canal was circumferentially divided from the puborectalis muscle and IAS, and then part of EAS was cut, if involved, after the skin around the anus was stretched by sutures to achieve an optimal view (Fig. 2d).
7. When head and tail dissection met, the tumor was removed via the anus, the specimen was cut with a linear stapler, and the DRM of the specimen was sent for intraoperative frozen resection (Fig. 2e, f).
8. The anus was dilated, and the radiation technician adjusted the INTRABEAM device at the same time.
9. When the results were returned, under the laparoscopic surveillance, the applicator was pushed to the tumor bed via the anus (Fig. 2g, h). The small intestine was moved in the cranial direction and was protected with wet gauze from irradiation. The ureters also are isolated with wet gauze (Fig. 2i).
10. After that, a single dose of 18 Gy of IORT was administered. When the IORT was complete, the hand-sewn colon-anal anastomosis and a prophylactic ileostomy were made simultaneously.

### Radiation dose

The INTRABEAM PRS can deliver a large dose (10–20 Gy) to the target area with rapid dose attenuation. In our study, according to the tumor and DMR results, a dose ranging from 16 to 18 Gy was chosen for INTRABEAM IORT.

### Chemotherapy regimen

After surgery, if the laboratory indicators such as white blood cell (WBC) counts had been qualified, patients were suggested to undergo the XELOX chemotherapy regimen (oxaliplatin and capecitabine) as soon as possible. Oxaliplatin was administered via intravenous infusion, at a dose of 130 mg/m^2^, for 3 h on the first day, and capecitabine was administered orally 2 times every day for 14 days, at a dose of 1000 mg/m^2^. The chemotherapy described above was repeated once every 3 weeks, and a total of 6–8 cycles was suggested.

## Results

The 12 patients, who included 9 male patients and 3 female patients, were preoperatively evaluated as having T3 or node-positive (T3N0M0, T1–3N+M0) cancer and underwent Lap ISR with INTRABEAM IORT (Table 1). The median age of the patients was 64.5 years (range 55–81 years) and the mean distance from the low edge of the tumor to the dentate line was 1.8 cm (range, 1.5–2.5 cm). Two patients had IAS invasion, while none had EAS invasion, and 5 patients had a positive circumferential resection margin (CRM) according to the preoperative MRI evaluation.

**Table 1:** The characteristics of patients

Characteristics	*N*
Gender	
F	3
M	9
Postoperative pathology stages	
T2	3
T3	9
Postoperative pathology lymph nodes	
Negative	6
Positive	6
Differentiation	
Well	1
Moderately	10
Moderately-poorly with mucinous	1
Tumor location (distance to DL)	
1–2 cm	7
2–3 cm	5
Chemotherapy after surgery	
Y	11
N	1

All patients had negative results from the intraoperative frozen resection. Postoperative pathology revealed that 9 patients had T3 tumors and 6 patients had node-positive tumors. The average number of harvested mesenteric lymph nodes was 16 (range, 10–18), and the mean number of harvested lymph nodes around the rectum was 15 (range, 9–19). The mean number of harvested inferior mesenteric artery root lymph nodes was 4 (range, 1–9), while none of the patients had positive nodes. The histopathologic types included moderately adenocarcinoma (10 cases), high-moderately adenocarcinoma (1 case), and moderately-poorly adenocarcinoma with mucinous adenocarcinoma (1 case). The mean bowel recovery time was 3 days (range, 2–5 days).

The mean radiation time was 27 min and 15 s, and the mean radiative dose was 17.3 Gy (range 16–18 Gy). Currently, the 16–18 Gy single dose used for Lap ISR is still under evaluation, and more factors will be considered with larger sample sizes and longer follow-up periods in future studies. The short-term outcomes, including acute radiation injury, complications, LR, function of the anus, and overall survival (OS), were assessed. Based on the Common Toxicity Criteria (CTC) of the Radiation Therapy Oncology Group (RTOG) [15], no acute kidney failure or acute radiation injury of the bladder or pelvis was observed at the initial 3-month follow-up. Until now, in our center, we have performed INTRABEAM IORT combined with the surgeries of Miles, Dixon, Hartmann, Lap ISR, and transanal TME (TaTME) in Primary locally advanced rectal cancer for more than 4 years, and no obvious radiative toxicity has occurred.

In our study, the LR was defined as any presence of anastomotic, pelvic, or lateral node recurrences recorded by pathologic or clinical examination, regardless of whether distant metastases occurred. The median follow-up time was 18.5 months (range, 3–45 months), and no one died. Until now, two patients including one male and one female patient experienced LR at the 13th month and 31st month, respectively. The male patient refused to receive the chemotherapy, and pulmonary metastases were found 15 months later. Furthermore, he experienced anastomotic stenosis, which was resolved with surgery 4 months postoperatively. The female patient with IAS invasion experienced distant metastases to the liver and gluteus maximus muscle at 35 months after IORT, and she received radioactive seeds (125I) for the hip metastases. One male patient experienced perianal abscess 5 months postoperatively, and it was resolved with surgery; thus, delayed stoma reversal was performed 1 year later. Ten patients received six cycles of the XELOX chemotherapy regimen (capecitabine with oxaliplatin) on schedule, and none of the other patients received additional radiotherapy postoperatively. The latest patient also has received one cycle of chemotherapy without radiotherapy on schedule. Eleven patients returned for an ileostomy, with a median time of 4.6 months (range, 3–12 months).

## Discussion

For locally advanced rectal cancer, total mesorectal excision (TME) has been leading to improved LR and OS [16]. As one of the key prognostic factors that determine the LR, CRM involvement is related to LR or the development of distant metastases. For patients with locally advanced low rectal tumor, the crucial purpose of treatment is not only the preservation of the anus but also the better local control. Thus, multiple modalities, involving surgery, EBRT, and chemotherapy, are still required, and T3 or node-positive (T3N0M0, T1–3N+M0) cancer patients are usually recommended to receive neoadjuvant radiotherapy preoperatively in order to achieve downstaging, and the rate of LR ranges from 6 to 10% after neoadjuvant therapy [17].

For the purpose of anal preservation and satisfactory postoperative quality of life, Lap ISR aims to reserve the levator ani muscle, EAS, and part of the IAS for defecation function, which greatly improves the quality of life and psychological state of patients. The EAS mostly accounts for generating squeeze pressure in the anus, and IAS is responsible for 70–85% of anal resting pressure [18]. Our ARM results illustrated that the postoperative resting pressure was evidently reduced, while the squeeze pressure was reduced slightly after the ileostomy reversal. Although the symptoms such as increased stool frequency and tenesmus occurred in our patients and affected life quality in an initial period of ileostomy reversal, with the higher Saito scores (Table 2), the patients were satisfied with the improved outcomes over time. The Wexner scores in patients, especially those with anastomotic stenosis, were poor during the early period but improved over time. Yokota et al. [19] reported that the Wexner scores recovered within 2 years in patients following ISR. Our outcomes showed that the recovery time ranged from 15 to 30 months and that long-term anal outcomes still need to be further assessed.

**Table 2:** The Saito functional questionnaire and Wexner score after ileostomy reversal

Variable	Post 1 months (*n* = 12)	Post 3 months (*n* = 11)
Stool frequency per 24 h	6.2 ± 1.8	4.3 ± 2.1
Urgency	9	6
Stool fragmentation	10	7
Dyschesia	3	1
Feces-flatus discrimination	7	4
Antidiarrheal medications	6	3
Dietary restriction	5	2
Pad	9	4
Wexner score	8.4 ± 4.2	7.6 ± 3.7

Compared with open ISR, Lap ISR provides a clear visualization for operative procedures, which can avoid the accidental damage to the hypogastric nerves, the ureter, and the pelvic plexus [20]. During open ISR, it is easy to damage the rectum or puborectalis muscle when removing the mesorectum and hiatal ligament due to the limited field of vision. However, Lap ISR can avoid the risks described above and can even allow separation of 1 cm down to the intersphincteric groove, which benefits the transanal dissection of the intersphincteric groove. Furthermore, with the use of the transanal approach for Lap ISR, it is easier to identify the resection of the DRM, and the risks of positive CRM are reduced under the direct surveillance. The fact that all patients in our study achieved R0 resection may owe to the cooperation of the two procedures.

Although the rate of LR of locally advanced rectal cancer has evidently decreased with the introduction of TME [21], Lee et al [22] reported that T3 patients had a worse 3-year disease-free survival (DFS) of 38% compared with other patients (T1, 84.8%; T2, 72.9%). IORT allows the precise delivery of a large tumoricidal dose to the target areas in order to reduce LR during surgery [23]. Compared with historical controls who did not receive IORT, patients with locally advanced rectal cancer who underwent IORT were reported to have higher OS and a lower rate of LR by Wallace et al. [24]. Cantero-Munoz et al. [25] reported a systematic review of 15 studies and revealed 5- to 6-year local control rates of > 80% and an OS of 65% for primary locally advanced rectal cancer patients treated with IORT.

Conventionally, for locally advanced rectal cancer advanced patients, especially those with stage T3 or T4 cancer, long-course radiotherapy (45 Gy in 25 fractions or 50.4 Gy in 28 fractions) or short-course radiation therapy (25 Gy in 5 fractions) is recommended. However, for IORT, the radiation dose of 18–20 Gy is equivalent to the external dose of 50 Gy [26]. As a novel mobile device, the INTRABEAM IORT has characteristics of a small high-physical dose and “sphere of equivalence,” which can generate isotropic dose distribution in the applicator with a large radiation dose (10–20 Gy) to the targeted area. This approach not only inhibits the potential proliferation or metastasis of residual tumor cells but also shortens the treatment time [27].

Currently, for locally rectal cancer advanced patients, especially T3 or T4 stage, long-course radiotherapy (45 Gy in 25 fractions or 50.4 Gy in 28 fractions) or short-course radiation therapy (25 Gy in 5 fractions) is recommended [28]. However, for IORT, the radiation dose of 18–20 Gy is equivalent to the external dose of 50 Gy [26].

In a multi-institutional phase randomized trial of IORT for locally advanced (T3 or T4 or N+, and M0) rectal cancer, Dubois et al. [29] delivered 18 Gy in the IORT arm and the results revealed that there was no significant superior radiative toxicity. In a study of INTRABEAM IORT in locally advanced or recurrent rectal cancer by Potemin et al. [30], a median surface dose of 14.8 Gy (range 9.4–17.0 Gy) was prescribed and no radiation-related events or complications were observed. Guo et al. [9] also delivered a median safe surface dose of 14.4 Gy (range 13.4–23.1 Gy) and a dose of 5 Gy was prescribed to a depth of 1 cm in locally advanced or recurrent rectal cancer with INTRABEAM IORT. Above all, in our study, a dose ranging from 16 to 18 Gy was chosen.

With the increased distance from the applicator surface, the dose of INTRABEAM PRS attenuates quickly so that it can lead to better local control without damage and long-term toxicity to adjacent critical structures, and the wet gauze we used to isolate the applicator and the adjacent critical organs further enhanced the efficacy. In addition, the applicator with a flexibility at 6 degrees of freedom [14] enabled it to be easily placed into the targeted area via the anus, which not only avoided an additional abdominal incision but also was in accordance with the concept of “Natural Orifice Transluminal Endoscopic Surgery (NOTES).”

After reviewing the literature, only two studies were found to have been published on the application of INTRABEAM IORT in the locally advanced or recurrent rectal cancer. Gou et al. [9] reported a retrospective review of 42 patients treated with INTRABEAM IORT, and the 1-year LR and distant metastasis rates were 16% and 32%, respectively, in the whole cohort. Potemin et al. [30] reported that the LR rate was 13% in 68 patients (47 stage II patients vs 21 stage III patients) treated with INTRABEAM IORT. The outcomes in our center revealed improved local control, and LR found in 2 patients suggests that postoperative chemotherapy is necessary and that higher doses of IORT (> 18 Gy) should be given in the patients with IAS invasion.

Our short-term outcomes also revealed very low risks of complications. It has been reported that 0.9–13% of Lap ISR patients experienced anastomotic leakage (AL) in the different studies, and the anastomotic stricture rate was higher in the AL group [31]. Recently, the incidence of anastomotic stricture after ISR has been reported to be from 0 to > 16% with no standard incidence set. During the operation, the color of the anal canal tissues near the anastomosis gradually changed to normal, which indicated a good blood supply.

In our study, two male patients experienced anastomotic stricture. One patient experienced perianal abscess and then underwent anastomotic stricture, and the other male patient experienced anastomotic stricture directly, while no female patients experienced anastomotic stricture. Both patients underwent anal dilation in the operative room, and the occurrence of perianal abscess and anastomotic stenosis might owe to the transanal hand-sewn coloanal anastomosis (HCAA). In a study of the Chinese population, Cong et al. [32] reported that 93 patients underwent ISR with HCAA and that 20 patients (21.5%) had AL.

To our knowledge, our study is the first to report the experience and short-term outcomes of patients with primary locally advanced low rectal cancer who underwent the Lap ISR and INTRABEAM IORT using low-energy X-rays, and several advantages of the treatment modality are considered as follows.

First, Lap ISR has benefits of preserving the anus and lowering the positive rate of DRM and CRM in high-risk patients, and the addition of INTRABEAM IORT using low-energy X-rays can further enhance the LC. Second, dose attenuation of INTRABEAM IORT can enhance the radiotherapy in the tumor bed while reducing injury to surrounding normal structures. Third, due to the mobility of the device, INTRABEAM IORT can be performed in the traditional operation room instead of the need for transferring patients to a specially shielded room, which not only shortens the operative time but also lowers the risk of transfer. Fourth, based on the concept of NOTES, the removal of the specimen and the input of the IORT applicator, which are both performed transanally, can avoid an additional abdominal incision, thus achieving good cosmetology.

Regardless of whether Lap ISR surgery or the IORT procedure is performed, we should pay attention to the preservation of anorectal function. The transanal approach for Lap ISR requires resection of part of the intersphincteric muscle [33], and radiotherapy may induce fibrosis around the rectum, thus affecting the compliance of the rectum [34]. Both of the procedures may lead to the low anterior resection syndrome (LARS), a complex of symptoms consisting of incontinence for flatus and/or feces, constipation, urgency, and bowel movements [35].

In our study, no symptoms of urinary dysfunction were observed. The results of the urinary function questionnaire were good, which indicated the good preservation of the automatic nerves in the manipulation of Lap ISR and the protection of INTRABEAM IORT. Therefore, in future follow-up studies, we should pay attention not only to local control, anastomotic stenosis, anorectal manometry, and incontinence but also to LARS and urethral function.

Although anal function was reduced and short-term complications such as perianal abscess and anastomotic stenosis occurred postoperatively, the Wexner and Saito scores improved over time, and patients were satisfied with the final outcomes of anal preservation. Furthermore, no acute radiation injury was observed in the short-term follow-up. Moreover, advantages such as higher dose homogeneity, omission of normal structures from the radiation area, and the acceptable outcome of anal preservation were proved. At present, the short-term outcomes are satisfying, and the long-term effects need to be further assessed.

However, limitations were evident due to the strict indications and the single center approach, the limited number of patients in the current study, and the fact that the study was a retrospective design instead of a randomized trial. Furthermore, the follow-up period for the entire group was relatively short. We hope that more evidence will support this novel treatment modality for primary locally advanced low rectal cancer as more patients are enrolled in future studies.

## Conclusions

For primary locally advanced low rectal cancer patients evaluated preoperatively with T3 or node-positive (T3N0M0, T1–3N+M0) tumors, our preliminary experience suggests that Lap ISR with INTRABEAM IORT using low-energy X-rays may provide a safe and feasible treatment modality for anal preservation and improved local control.

## Data Availability

The datasets used and/or analyzed during the current study are available from the corresponding author on reasonable request.

## Abbreviations

CRM: Circumferential resection margin
DFS: Disease-free survival
DRM: Distal resection margin
EBRT: External beam radiotherapy
IORT: Intraoperative radiotherapy
LC: Local control
NOTES: Natural orifice transluminal endoscopic surgery
OS: Overall survival
TaTME: Transanal total mesorectal excision
